# Persistence within dendritic cells marks an antifungal evasion and dissemination strategy of *Aspergillus terreus*

**DOI:** 10.1038/s41598-017-10914-w

**Published:** 2017-09-06

**Authors:** Shih-Hung Hsieh, Oliver Kurzai, Matthias Brock

**Affiliations:** 1 Fungal Genetics and Biology Group, School of Life Sciences, University of Nottingham, University Park, NG7 2RD Nottingham, UK; 2 Microbial Biochemistry and Physiology, Leibniz Institute for Natural Product Research and Infection Biology – Hans-Knoell-Institute, Adolf-Reichwein-Str. 23, 07745 Jena, Germany; 3 Institute for Hygiene and Microbiology, Medical Microbiology and Mycology, Julius-Maximilians-University Würzburg, Josef-Schneider-Str. 2, 97080 Würzburg, Germany; 4 Septomics Research Center, Leibniz Institute for Natural Product Research and Infection Biology – Hans-Knoell-Institute, Adolf-Reichwein-Str. 23, 07745 Jena, Germany

## Abstract

*Aspergillus terreus* is an airborne human fungal pathogen causing life-threatening invasive aspergillosis in immunocompromised patients. In contrast to *Aspergillus fumigatus*, *A. terreus* infections are associated with high dissemination rates and poor response to antifungal treatment. Here, we compared the interaction of conidia from both fungal species with MUTZ-3-derived dendritic cells (DCs). After phagocytosis, *A. fumigatus* conidia rapidly escaped from DCs, whereas *A. terreus* conidia remained persisting with long-term survival. Escape from DCs was independent from DHN-melanin, as *A. terreus* conidia expressing *wA* showed no increased intracellular germination. Within DCs *A. terreus* conidia were protected from antifungals, whereas *A. fumigatus* conidia were efficiently cleared. Furthermore, while *A. fumigatus* conidia triggered expression of DC activation markers such as CD80, CD83, CD54, MHCII and CCR7, persistent *A. terreus* conidia were significantly less immunogenic. Moreover, DCs confronted with *A. terreus* conidia neither produced pro-inflammatory nor T-cell stimulating cytokines. However, TNF-α addition resulted in activation of DCs and provoked the expression of migration markers without inactivating intracellular *A. terreus* conidia. Therefore, persistence within DCs and possibly within other immune cells might contribute to the low response of *A. terreus* infections to antifungal treatment and could be responsible for its high dissemination rates.

## Introduction

Invasive fungal infections cause major health problems especially in critically ill and immunocompromised patients. It has been estimated that *Candida*, *Aspergillus*, *Cryptococcus* and *Pneumocystis* species cause about two million infections each year^1^. Patients at risk for invasive aspergillosis frequently suffer from haematological malignancies, chronic lymphoproliferative disorders or underwent allogeneic stem cell or solid organ transplantation^2^. As *Aspergillus* species are ubiquitous in the environment and produce airborne conidia, the lung is the primary target organ through inhalation of conidia. Although *Aspergillus fumigatus* contributes to 68–72% of cases of invasive aspergillosis and is therefore the major pathogenic species^2, 3^, *Aspergillus terreus* is responsible for 3–12% of all cases and numbers have been increasing in recent years^4, 5^. In contrast to *A. fumigatus*, invasive infections by *A. terreus* show high rates of dissemination to secondary organs^6^ and, despite the use of recommended therapeutic approaches, invasive aspergillosis by *A. terreus* only shows poor response to antifungal treatment^4^. Therefore, mortality rates from *A. terreus* infections are even higher than those caused by other *Aspergillus* species^4^.

The innate immune system plays an important role in clearing *Aspergillus* infections^7^. After entering the respiratory tract, *Aspergillus* conidia are recognized by various pattern recognition receptors, such as C-type lectin receptors, or toll-like receptors^7, 8^, which are widely expressed on the surface of immune cells^8, 9^. Several studies have shown that alveolar macrophages and neutrophils are essential immune cells in orchestrating the immune response, controlling and eliminating *Aspergillus* conidia^7–9^. However, some striking differences in conidia interactions of *A. fumigatus* and *A. terreus* with immune cells have been observed^10^. Once phagocytosed by macrophages, the dihydroxynapthalene-melanin (DHN-melanin) of *A. fumigatus* conidia inhibits phagolysosome acidification and allows conidia germination and escape from macrophages^10, 11^. In contrast, *A. terreus* conidia produce a different type of melanin, the so-called Asp-melanin, which protects conidia from UV radiation and reduces phagocytosis by amoeba^12^, but does not inhibit acidification of macrophage phagolysosomes^10^. Consequently, *A. terreus* conidia reside in the acidic environment of phagolysosomes, in which they show long-term persistence without inactivation. This long-term persistence was further confirmed by *in vivo* studies using a murine inhalation infection model^13^. Mice treated with high doses of corticosteroids were susceptible not only to *A. fumigatus*, but also to *A. terreus* infections. Bioluminescence imaging revealed that all mice infected with *A. terreus* conidia developed initial invasive disease, but only about 50% of mice succumbed to infection. Survivors showed no clinical signs of invasive disease at day 14 post infection. However, lung sections indicated the presence of conidia and plating of lung homogenates revealed that these conidia were still viable^13^. Furthermore, when immunocompetent mice were infected with *A. terreus* conidia, only a transient pro-inflammatory response was detected, which implied that infection was rapidly cleared. However, histopathology at days 1, 3 and 5 after infection revealed significant amounts of intracellular conidia and determination of colony forming units showed that these conidia were still viable^10^. This indicates that persistence within immune cells is not only observed under immunocompromised conditions, but also in the immunocompetent situation. However, the subtypes of immune cells in which *A. terreus* conidia are able to persist have not been investigated yet.

Besides macrophages, dendritic cells (DCs) are another type of professional phagocytic cells that migrate to regional and draining lymph nodes where they present fungal antigens to T-cells and prime T-cell immune responses^14, 15^. Furthermore, some fungal pathogens, such as *Histoplasma capsulatum* are more efficiently restricted in the conidia to yeast transformation by DCs compared to macrophages, indicating an additional role of DCs in the control of fungal infections^16^. *A. fumigatus* conidia are efficiently phagocytosed by DCs *via* mannan receptors and C-type lectins of galactomannan specificity^17^. While a short-term interaction with DCs does not inactivate *A. fumigatus* conidia, three days after infection pulmonary DCs had transported conidia to draining lymph nodes and spleen without signs for viable fungal cells. This indicates the general ability of DCs to inactivate and degrade *Aspergillus* conidia for efficient antigen presentation^17^. Furthermore, it has been shown that DC recruitment in neutropenic mice increases during *A. fumigatus* infection and a depletion of DCs under this setting further impairs fungal clearance^18^. However, effective maturation of dendritic cells requires the interaction with neutrophils that stimulate DC maturation and migration to draining lymph nodes^19^. It has also been shown that both *A. fumigatus* and *A. terreus* infections trigger Th17/Th1 immune responses^20^. However, while *A fumigatus* also elicited Th2 activating cytokine production after infection, a Th2 immune response with *A. terreus* conidia has not been observed^20^. Th2 cytokines are promoted by IL-4 and IL-10 that are secreted by DCs mainly in response to hyphal growth, but expression of these cytokines is not promoted in *A. terreus* infections^10^. This finding suggests that *A. terreus* conidia may persist in DCs without germination and escape as observed in previous studies on macrophages^10^.

Taking into account that *A. terreus* infections are often refractory to antifungal treatment ^4^, show long term persistence in infected murine lungs^10, 13^ and display high dissemination rates in human patient populations^6^, we compared the interaction of *A. terreus* and *A. fumigatus* conidia with a human dendritic cell line. Our results suggest that *A. terreus* might hitchhike DCs for dissemination and gets protected from antifungal treatment.

## Results

### Selection of dendritic cells for interaction studies

To study the interaction of *A. fumigatus* and *A. terreus* conidia with dendritic cells we tested the suitability of the CD34^+^ human acute myeloid leukaemia cell line MUTZ-3^21^ as an alternative to monocyte-derived human dendritic cells (moDCs) as MUTZ-3 cells have been shown to display the full range of functional antigen processing and presentation^21^. Similar to the differentiation of CD1a-positive and CD14-negative moDCs from peripheral blood monocytes after stimulation with human recombinant GM-CSF and IL-4 (Fig. S1A), MUTZ-3 cells were reproducibly differentiated into immature DCs (iDCs) when incubated in the presence of human recombinant GM-CSF and IL-4^22^ (Fig. S1B). Therefore, subsequent studies were performed with MUTZ-3 cells differentiated into iDCs and relevant data were confirmed by the use or primary moDCs.

### Phagocytosis of resting *A. fumigatus* and *A. terreus* conidia by iDCs

**Figure 1:**
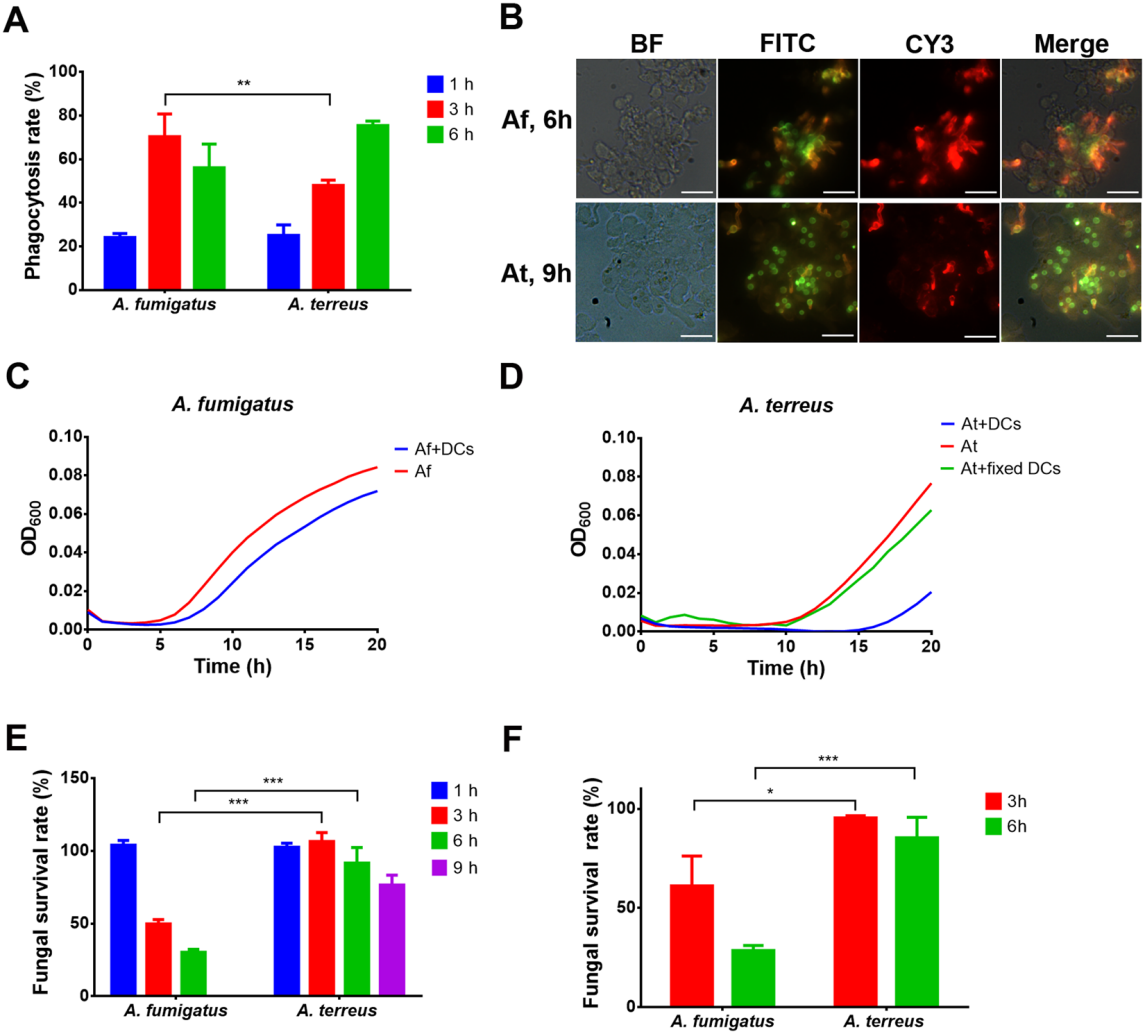
Phagocytosis, survival and escape of *A. terreus* and *A. fumigatus* in the interaction with DCs. (**A**) Phagocytosis of *Aspergillus* conidia by MUTZ-3 derived iDCs. Phagocytosis rate was determined after 1 h, 3 h and 6 h of confrontation from about 1000 conidia that co-localised with DCs. (**B**) Microscopic visualisation of phagocytosis of *A. fumigatus* conidia at 6 h and *A. terreus* conidia at 9 h. FITC-labelled conidia were applied to DCs. Red Cy3-staining indicates fungal elements with contact to medium. Only extracellular *A. terreus* conidia form germ tubes as indicated by co-localisation of FITC and Cy3. Scale bar = 20 µm; BF, bright field. (**C**) Growth analysis of *A. fumigatus* conidia incubated in the presence and absence of DCs. (**D**) Growth analysis of *A. terreus* conidia incubated in the absence or presence of viable or fixed DCs. (**E**) Survival of *A. fumigatus* (1, 3, 6 h) and *A. terreus* (1, 3, 6, 9 h) conidia in co-incubation with MUTZ-3 derived iDCs. (**F**) Survival of *A. fumigatus* and *A. terreus* conidia (both at 3 h and 6 h) in co-incubation with moDCs. All data represent mean values obtained from three independent experiments. Data in (A) show mean values + SD. Statistical analysis was carried out by two-tailed Student’s t-test (*p < 0.05; **p < 0.01; ***p < 0.005).

Previous *in vitro* studies showed that *A. terreus* conidia were rapidly phagocytosed by macrophages and remained resting in acidified phagolysosomes, whereas *A. fumigatus* conidia suppressed acidification and escaped by the formation of germ tubes^10^. When confronted with MUTZ-3 iDCs, 70% of *A. fumigatus* conidia were phagocytosed within 3 h and this rate declined with prolonged incubation time (55% at 6 h; Fig. 1A). This appeared mainly due to rapid germ tube formation and escape from DCs (Fig. 1B) and was in line with phagocytosis rates observed with murine fetal skin-derived DCs that showed about 70% internalization of *A. fumigatus* conidia after two hours with a decreasing rate after 4 h of confrontation^17^. On the contrary, despite higher galactomannan and β-glucan surface presentation of *A. terreus* conidia compared to *A. fumigatus* ^10^, phagocytosis rate of *A. terreus* conidia was slightly delayed with 48% internalisation at 3 h. However, phagocytosis rate increased to about 75% at 6 h without significant germ tube formation of intracellular conidia (Fig. 1A). Microscopic analyses revealed some germination of *A. terreus* conidia after 9 h, but this germination was mainly restricted to conidia that had not been internalised by DCs (Fig. 1B). Intracellular conidia at this 9 h time point were either resting or showed some initial swelling. This implied that *A. terreus* conidia, once phagocytosed by iDCs do not tend to rapidly escape. These obvious differences in phagocytosis and fungal escape tempted us to determine growth rates of *A. fumigatus* and *A. terreus* conidia in the presence and absence of dendritic cells. During co-incubation with iDCs *A. fumigatus* showed a delay in the increase of optical density by only about 2 h compared to conidia grown in the absence of iDCs (Fig. 1C), which is in agreement with the rapid germ tube formation observed by microscopic analyses. In contrast, while *A. terreus* conidia generally tend to germinate slower than those from *A. fumigatus*, germination in the presence of DCs was further delayed by at least 5 h (Fig. 1D) and most likely derived from conidia not phagocytosed by DCs. This delay was strictly dependent on the presence of viable DCs, as *A. terreus* germination speed was not delayed when conidia were incubated in the presence of fixed DCs (Fig. 1D).

### Survival of *A. terreus* and *A. fumigatus* in confrontation with DCs

Due to the low rate of intracellular germination of *A. terreus* conidia inside DCs, viability was determined by analysing the number of colony forming units (CFU) after lysis of DCs. Although *A. fumigatus* escaped DCs and displayed 100% viability after one hour of confrontation with MUTZ-3 iDCs, a significant reduction in viable counts were observed at three and six hours (Fig. 1E). However, due to the formation of dense mycelium from extracellular hyphae, no CFU determination at later time points was possible. In contrast to *A. fumigatus*, conidia of *A. terreus* were more resistant against DC-mediated killing. Survival rate of *A. terreus* conidia remained at about 100% after 3 h and only slightly dropped down to about 70–80% at 9 h (Fig. 1E). When experiments were repeated with human monocyte-derived moDCs comparable results as for MUTZ-3 iDCs were obtained for both fungi (Fig. 1F). This confirms the suitability of the MUTZ-3 cell line in analysing interactions of *Aspergillus* conidia with DCs and furthermore reveals that *A. terreus* conidia remain persistent and viable within DCs.

### Analysis of persistence *versus* escape strategies

**Figure 2:**
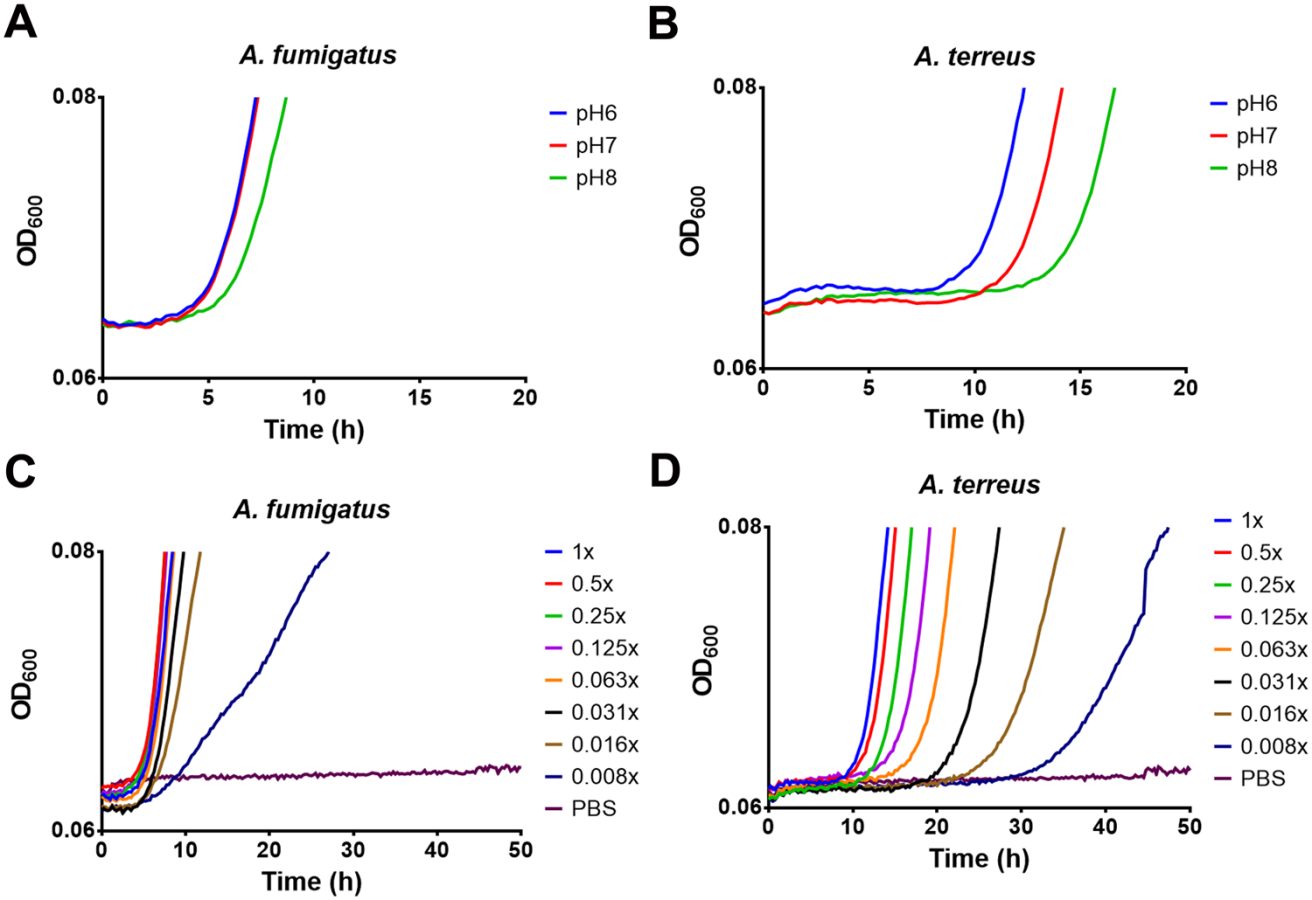
Germination of *Aspergillus* conidia in dependence of environmental pH and nutrient availability. (**A**,**B**) Effect of medium pH on germination of *A. fumigatus* (**A**) and *A. terreus* (**B**). (**C**,**D**) Germination of *A. fumigatus* (**C**) and *A. terreus* (**D**) conidia in dependence on nutrient availability. RPMI/glucose medium was serially diluted and growth was monitored as increase in optical density at 600 nm (OD_600_) using a microplate reader. Growth curves in (**A**–**D**) result from two independent experiments each containing three technical replicates.

Macrophages acidify phagolysosomes by an active proton pumping mechanism to effectively degrade foreign antigens. *A. fumigatus* inhibits this acidification by DHN-melanin that coats conidia^11^ and contributes to survival within and rapid escape from macrophages. On the contrary, *A. terreus* conidia produce Asp-melanin^12^, which does not inhibit phagolysosme acidification and, consequently, conidia reside within acidified phagolysosomes^10^. However, it has been shown that the escape of *A. terreus* conidia from macrophages can be stimulated by either inhibiting the V-H^+^-ATPase with Bafilomycin or by recombinant expression of the *Aspergillus nidulans* naphthopyrone synthase gene *wA*, which is responsible for production of a precursor molecule of DHN-melanin^10^. However, when we confronted *A. terreus* conidia expressing the *wA* gene with iDCs, germination showed the same delay (Fig. S2) as wild-type *A. terreus* conidia (Fig. 1D). This indicates that the different type of melanin formed by *A. terreus* conidia is not the direct cause for persistence in DCs. Furthermore, lysotracker staining of DCs after phagocytosis of either *A. fumigatus* or *A. terreus* conidia did not indicate acidified phagolysosomes with either species (Fig. S3). This is in agreement with an active NOX2 dependent alkalinisation mechanism in the phagolysome membrane of DCs and is important for antigen cross-presentation and leads to a phagolysosome pH of about 8^23^. This confirmed that an acidic environment is not the cause of delayed germination of *A. terreus* conidia. However, when we investigated the effect of environmental pH on germination of conidia, only a minor delay of germination was observed with *A. fumigatus* conidia at pH 8 compared to pH 6 and 7 (Fig. 2A), whereas a delay of about three hours at a shift from pH 6 to 7 and a 6 h delay at a shift to pH 8 was observed with *A. terreus* conidia (Fig. 2B). Therefore, an alkaline environment might be one reason for trapping *A. terreus* conidia in DCs. However, an additional effect on inhibition of germination might derive from nutrient restriction within phagolysosomes. To test this assumption, we studied the germination of *A. fumigatus* and *A. terreus* conidia in serial dilutions of RPMI/glucose medium. Indeed, *A. fumigatus* conidia efficiently germinated without delay when RPMI/glucose medium was diluted by at least 64-fold (Fig. 2C), whereas germination speed of *A. terreus* conidia was strictly dependent on the concentration of nutrients (Fig. 2D). A subsequent investigation on *A. terreus* conidia germination in dependence on the limitation of specific nutrient sources indicated that either glucose or nitrogen limitation caused a delay in germination and limitation of both showed a cumulative effect (Fig. S4). As *A. terreus* and *A. fumigatus* both grow on minimal media, the overall nutritional requirements for both fungi appear to be similar. However, due to the unrestricted fast germination of *A. fumigatus* even at very low nutrient concentrations, the required nutrient threshold triggering germination seems much lower for *A. fumigatus* than for *A. terreus*. In conclusion, alkalinisation of phagolysosomes accompanied by a restricted nutrient supply traps *A. terreus* conidia in a resting state, whereas *A. fumigatus* efficiently germinates at pH 8 and shows rapid germination as soon as nutrients exceed a certain threshold value.

### Efficacy of antifungals on persisting intracellular conidia

**Figure 3:**
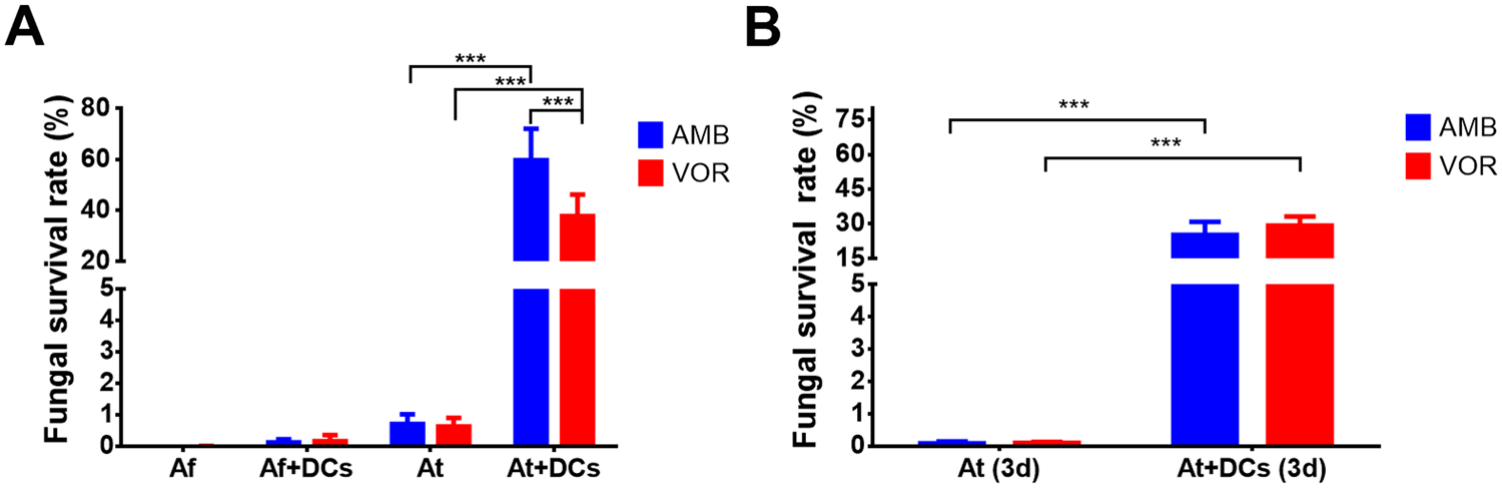
Effect of antifungals on survival of *Aspergillus* conidia in the presence of iDCs. (**A**) Effect of amphotericin B (AMB, 10 µg/ml) or voriconazole (VOR, 10 µg/ml) on survival of *A. fumigatus* and *A. terreus* conidia in the presence or absence of MUTZ-3-derived iDCs. Conidia were pre-incubated with DCs for 6 h before addition of antifungals to avoid inactivation of conidia prior to phagocytosis. DCs were lysed after 24 h for determination of CFUs. (**B**) Analysis of long-term persistence of *A. terreus* conidia in MUTZ-3-derived iDCs in the presence of antifungals. DCs were lysed after 72 h of incubation and CFUs were determined. All data show mean values + SD from three independent experiments. Statistical significance was calculated by using the two-tailed Student’s t-test (***p < 0.005).

Alkaline pH and nutrient limitation appear as major reasons for trapping *A. terreus* but not *A. fumigatus* conidia in a resting intracellular state. From this result two questions arose: (i) are conidia persisting in DCs protected from environmental stressors such as antifungals and (ii) how long can conidia survive within DCs? To address these questions we selected the antimycotics amphotericin B (AMB) and voriconazole (VOR) that were used in a concentration of 10 µg/ml, which is far above the MIC values of both drugs for the selected strains with about 0.5 µg/ml (AMB) and 0.25 µg/ml (VOR) for *A fumigatus* and 1 µg/ml (AMB) and 0.25 µg/ml (VOR) for *A. terreus* as determined by broth dilution tests. Therefore, we expected that all extracellular fungi that had not been phagocytosed or escaped from DCs were inactivated by the antifungals. Indeed, when conidia were incubated without iDCs in the presence of these antimycotics and analysed for CFUs after 24 h, about 100% of *A. fumigatus* conidia and about 99.5% of *A. terreus* conidia were inactivated with either drug (Fig. 3A), showing that the selected concentrations of both antimycotics were highly effective. Subsequently, *A. fumigatus* and *A. terreus* conidia were first pre-incubated for six hours in the presence of iDCs to allow for phagocytosis of extracellular conidia before antimycotics were added and CFUs were analysed after 24 h (Fig. 3A). Only about 1% of *A. fumigatus* conidia remained viable after this procedure, indicating that conidia that escaped DCs were inactivated by extracellular drugs and conidia that failed to escape were inactivated by DCs. In contrast, in the presence of DCs about 60% of *A. terreus* conidia survived AMB and 40% the VOR treatment. This implies that intracellular conidia were well protected from antifungal treatment and not efficiently inactivated by DCs. To test for a long-term persistence of *A. terreus* within DCs the experiment was repeated, but analysis of CFUs was performed after three days of incubation (Fig. 3B). While about 99.9% of free extracellular conidia were inactivated by either drug, 30% viable conidia were still re-isolated from DCs under either treatment. This experiment confirmed both assumptions from above: *A. terreus* conidia show an intracellular long-term persistence within DCs, which is accompanied by a protection from antifungal treatment.

### DC activation in the presence of *A. fumigatus* and *A. terreus* conidia

**Figure 4:**
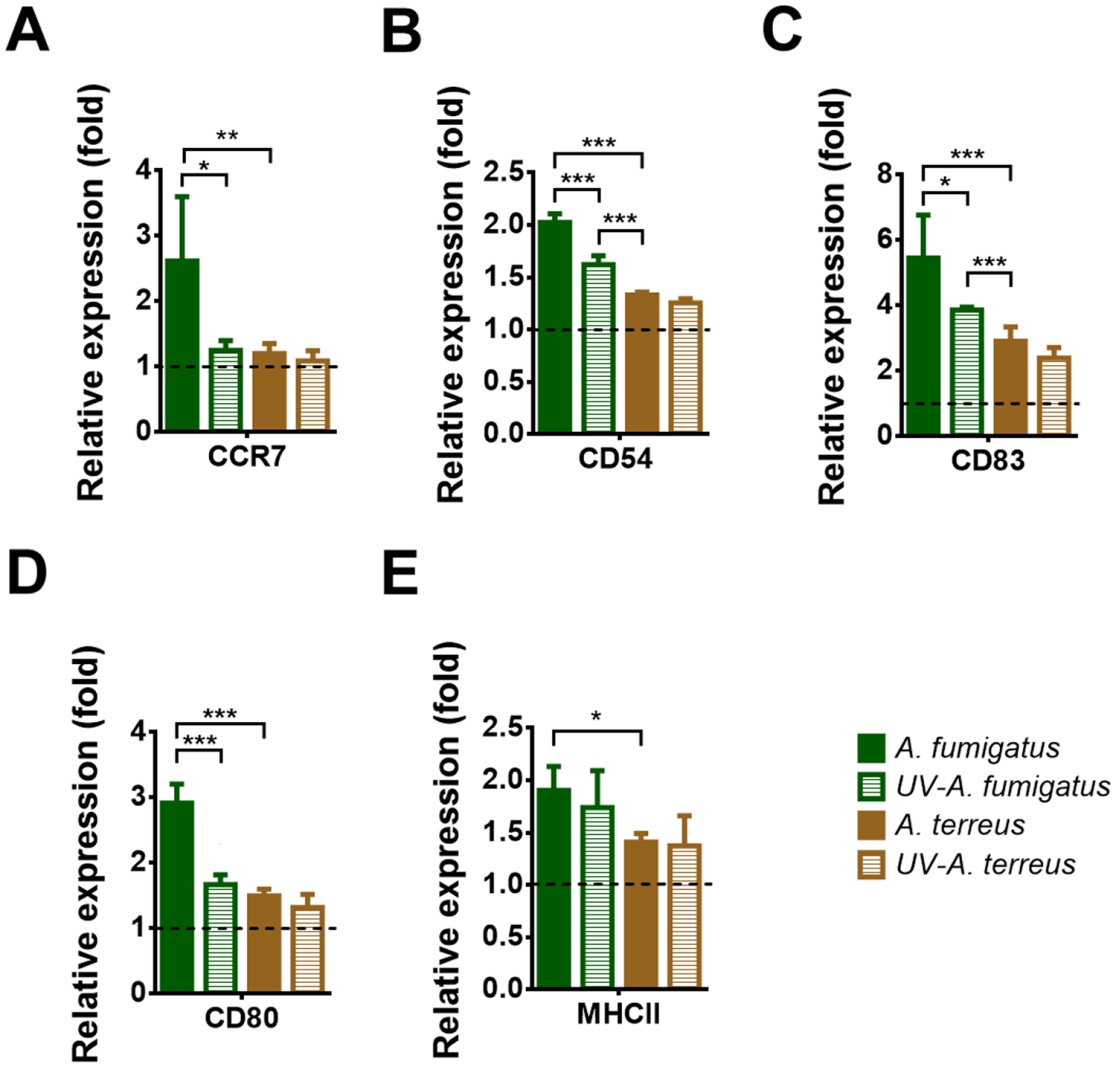
Expression of DC activation markers after confrontation with *A. fumigatus* and *A. terreus* conidia. DCs were pre-incubated for 6 h with UV-inactivated or viable *A. fumigatus* or *A. terreus* conidia before amphotericin B (2 μg/ml) was added to inhibit extracellular fungal growth. Expression levels were normalised to expression levels of uninfected DCs (dashed line). Marker expression was determined after a total incubation time of 24 h. (**A**) CCR7, (**B**) CD54, (**C**) CD83, (**D**) CD80 and (**E**) MHC II (HLA-DR). Data represent mean values + SD from three independent experiments. Statistics were performed by one-way ANOVA (*p < 0.05, **p < 0.01, ***p < 0.005).

Activation of DCs is required to trigger an adaptive immune response against *Aspergillus* infections^24^. In this respect, activation of the transmigration marker CCR7 is of special importance as it allows migration of DCs to lymph nodes for T-cell interaction and activation during *Aspergillus* infection^24^. Since *A. terreus* conidia showed a persistent phenotype within DCs, we wondered whether these conidia trigger activation and maturation of iDCs. Therefore, we first determined expression of the activation markers CCR7, CD83, CD80 and MHCII after short term incubation (6 h) with viable or UV-inactivated *A. fumigatus* and *A. terreus* conidia. Only the confrontation with viable *A. fumigatus* conidia resulted in a significant induction of CCR7 expression accompanied by induction of the activation marker CD83 and a similar trend was observed by either using MUTZ-3 DCs or moDCs (Fig. S5). UV-inactivated *A. fumigatus* conidia as well as viable or UV-inactivated *A. terreus* conidia did not induce CCR7 expression, but slightly induced expression of CD83 (Fig. S5C). Since maturation of DCs may take longer than 6 h especially when no other co-stimulatory cytokines or interactions with immune cells such as neutrophils are present^19^, we aimed in the characterisation of DC activation at later time points. To avoid complete destruction of DCs by extracellular hyphae, we added AMB after 6 h of confrontation, but lowered AMB concentration to 2 µg/ml to avoid perturbance of dendritic cell function. Such treatment has previously been shown not to grossly affect response of human DCs in the interaction with *A. fumigatus* conidia^24^. Activation marker expression was studied after a further incubation of 18 h and was normalised to cells treated with AMB but not confronted with conidia. As expected^24^, CCR7 and to a minor extend CD62L (not shown) as well as CD83, CD80, CD54 and MHCII were strongly expressed on DCs confronted with viable *A. fumigatus* conidia (Fig. 4). This indicates that these DCs matured and became activated for transmigration to lymph nodes through expression of CCR7 for stimulation of naïve T-cells. In contrast, while CD83, CD80, CD54 and MHCII, though to a lower extent, were also upregulated by UV-inactivated *A. fumigatus* and both, viable and UV-inactivated *A. terreus* conidia, no significant induction of CCR7 surface expression was observed for those pathogen preparations (Fig. 4). These partially activated DCs are assumed not to produce T-cell activating cytokines or stimulate immunogenic T-cells^25^.

### Cytokine production of DCs in response to *A. fumigatus* and *A. terreus* conidia

**Figure 5:**
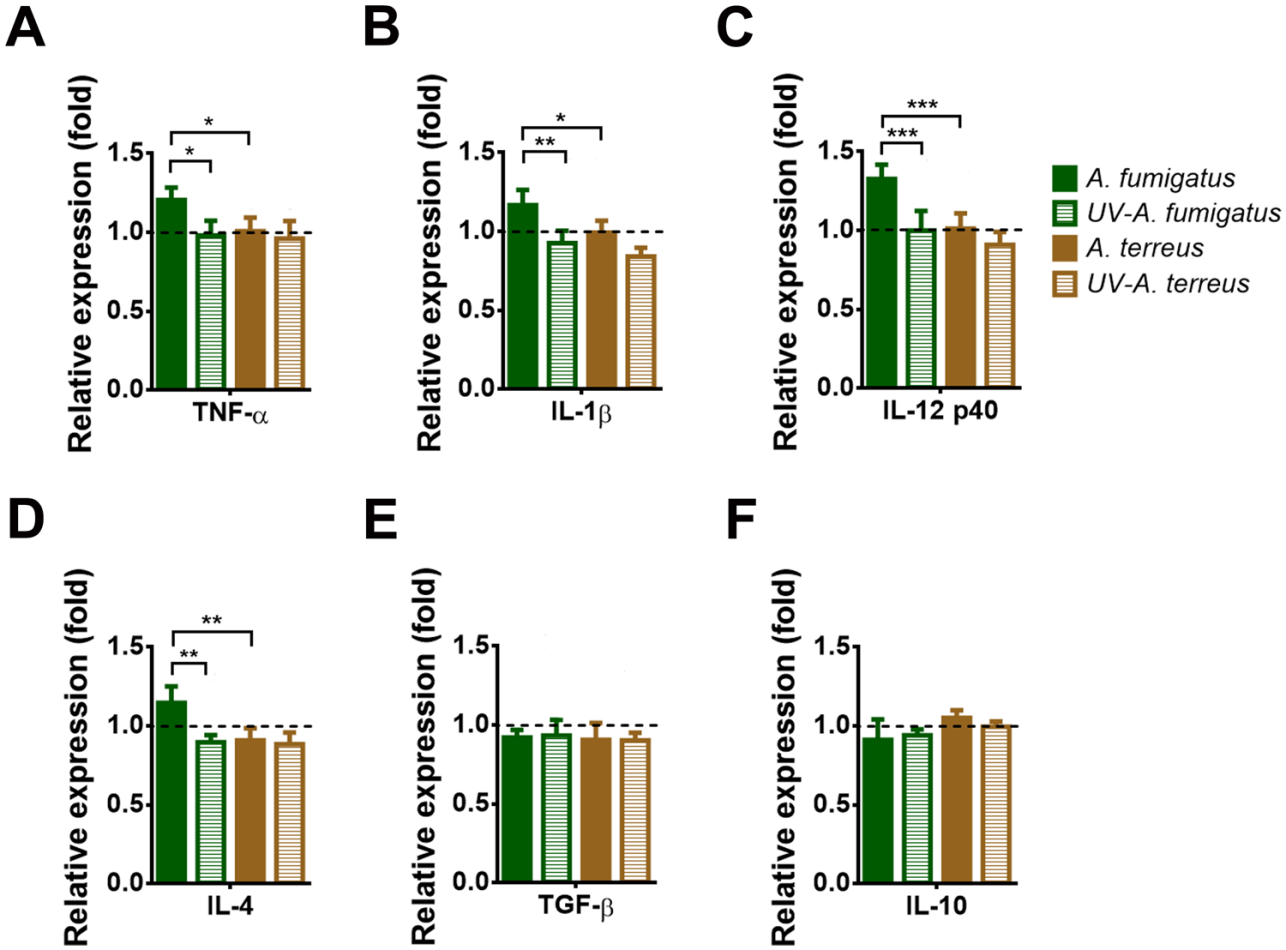
Cytokine production by DCs confronted with *A. fumigatus* and *A. terreus* conidia. MUTZ-3-derived iDCs were pre-incubated for 6 h with UV-inactivated or viable *A. fumigatus* or *A. terreus* conidia before amphotericin B (2 μg/ml) was added to inhibit extracellular fungal growth. Brefeldin A was added after a total of 24 h and cytokine production was determined 6 h later. Cytokine expression was normalised to expression levels of uninfected DCs (dashed line). (**A**) TNF-α, (**B**) IL-1β, (**C**) IL-12/IL-23 p40, (**D**) IL-4, (**E**) TGF-β and (**F**) IL-10 production. Bar diagrams indicate mean values + SD from three independent experiments. Statistics were performed by using one-way ANONA (*p < 0.05, **p < 0.01, ***p < 0.005).

From analyses of activation markers we assumed that only DCs confronted with viable *A. fumigatus* conidia ﻿are stimulated to produce cytokines that are critical to elicit innate and adaptive immune responses. Therefore, we analysed Th1, Th2, immunosuppressive and pro-inflammatory cytokine production of DCs in response to viable and UV-inactivated *A. fumigatus* and *A. terreus* conidia after 24 h of confrontation (Fig. 5). When incubated with viable *A. fumigatus* conidia DCs produced pro-inflammatory cytokines, such as IL-1β and TNF-α as well as T-cell stimulating IL-12 (Th1) and IL-4 (Th2) cytokines. In contrast, neither confrontation with UV-inactivated *A. fumigatus* nor viable or UV-inactivated *A. terreus* conidia resulted in a significant cytokine response of DCs (Fig. 5A-D), whereby the lack of cytokine production was not mediated by production of the immunosuppressive cytokines TGF-β and IL-10 (Fig. 5E-F). Therefore, we conclude that persisting *A. terreus* conidia do not induce a strong pro-inflammatory or immune regulatory response in DCs.

### Effect of TNF-α stimulation on DCs confronted with *A. terreus* conidia

**Figure 6:**
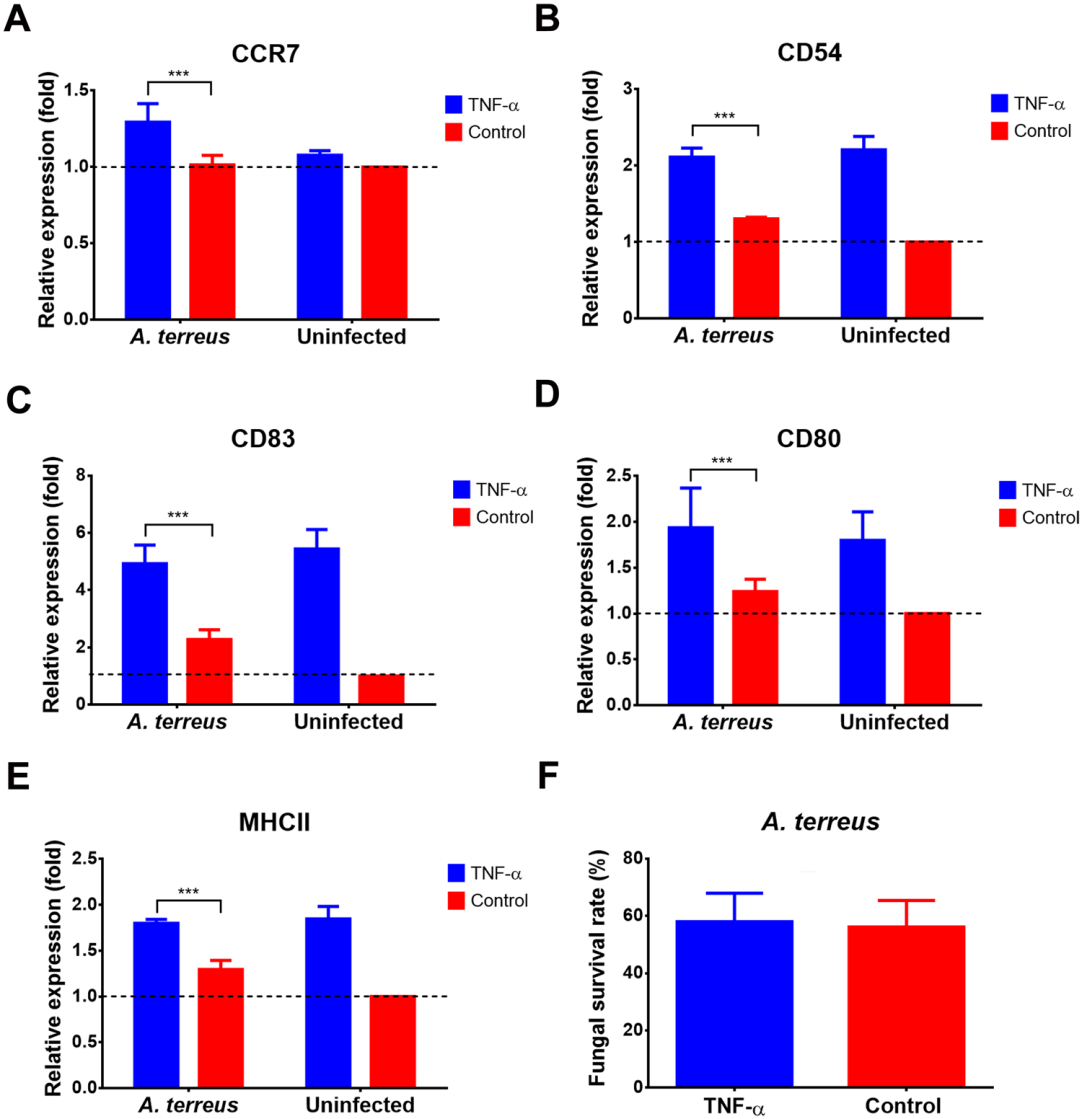
Effect of extracellular TNF-α on DCs containing persisting *A. terreus* conidia. MUTZ-3-derived iDCs were pre-incubated for 6 h with *A. terreus* conidia before amphotericin B (2 μg/ml) was added. Medium was either supplement with 100 ng/ml TNF-α or left untreated (control). Cells were incubated for a further 18 h before expression of (**A**) CCR7, (**B**) CD54, (**C**) CD83, (**D**) CD80 and (**E**) MHC II was analysed by flow cytometry. Data were normalised to expression levels from uninfected DCs without TNF-α supplementation (dashed line). (**F**) Survival of persisting *A. terreus* conidia under TNF-α treatment. Cells were lysed after 24 h and CFUs were determined. Statistics were performed by using the two-tailed Student’s t-test. All data represent mean values + SD from three independent experiments (***p < 0.005).

Our analyses revealed that *A. terreus* conidia persist within DCs, but neither trigger a significant cytokine response (Fig. 5) nor stimulate the expression of the transmigration markers CCR7 and CD62L (Fig. 4). However, due to their ability to transmigrate for antigen presentation, DCs appear to depict ideal candidates for transporting *A. terreus* to different body sites. This is of special interest as clinical reports have indicated that aspergillosis caused by *A. terreus* is frequently associated with high dissemination rates to secondary organs^6^. Previous studies showed that corticosteroid treated mice infected with high doses of *A. terreus* conidia display elevated levels of TNF-α in lung tissues, which might results from the germination of extracellular conidia^13^. Therefore, we stimulated DCs containing persisting *A. terreus* conidia with 100 ng/ml TNF-α and inhibited extracellular growth of *A. terreus* conidia by amphotericin B (Fig. 6). Indeed, TNF-α not only significantly stimulated the expression of the activation markers CD83, CD80, CD54 and MHCII, but also of the transmigration marker CCR7, indicating that TNF-α led to a full maturation of iDCs (Fig. 6A-E). However, when the intracellular survival of *A. terreus* conidia was investigated after 24 h, about 60% of conidia remained viable and this viability was undistinguishable from iDCs that were not stimulated by TNF-α (Fig. 6F). Therefore, TNF-α stimulation induces migration of DCs harbouring persisting *A. terreus* conidia and, therefore, might support fungal dissemination.

## Discussion

Invasive fungal infections remain a great challenge in critically ill patients and especially patients suffering from haematological malignancies are highly susceptible for invasive aspergillosis^2^. As *A. fumigatus* is most frequently re-isolated from patients with invasive aspergillosis, studies on immune interactions have strongly focused on this species. However, we have previously shown that macrophage interaction of *A. terreus* is significantly different from that observed with *A. fumigatus* ^10^. While *A. fumigatus* inhibits phagolysosome acidification by its DHN-melanin layer and rapidly germinates and pierces the phagocytic cell, *A. terreus* has been shown to reside in acidified phagolysosomes^10^. Persistence within macrophages is at least partially due to the different type of melanin produced by *A. terreus* ^12^, as expression of the naphtopyrone synthase *wA* increased *A. terreus* germination rates. However, as DCs do not tend to acidify their phagolysosomes^23^, it is well conceivable that the type of melanin does not influence persistence and escape in this type of immune cells and, in agreement, the expression of *wA* in *A. terreus* conidia did not trigger the escape from DCs.

Our analyses on the reasons why *A. terreus* conidia remain dormant within DCs indicated that both, the alkaline pH in DC phagolysosomes as well as restriction from nutrient supply significantly delays germination of *A. terreus* conidia. Strikingly, germination rates of *A. fumigatus* conidia were quite similar in a range from pH 6 to 8 and even at a very limited nutrient supply germination was initiated without any significant delay. Therefore, while the nutritional supply in DCs seems to exceed the required threshold value for germination of *A. fumigatus* conidia, the phagolysosomal environment forms a natural barrier for *A. terreus* germination and indicates that DCs do not need to produce specific growth inhibitory factors to control *A. terreus* germination. However, since *A. terreus* conidia can be stored for several weeks in nutrient limited solutions such as phosphate-buffered saline or water without significant loss in viability, they easily survive the nutrient limited environment within phagolysosomes. In addition, as conidia do not tend to germinate under these conditions, they may be treated as inactive antigen particles that induce expression of activation markers on dendritic cells without stimulating maturation by expression of the transmigration marker CCR7^24^. Unfortunately, these activated but not matured DCs may act as negative regulators of T-cell immune response. Interaction of T-cells with these so-called steady-state DCs inhibits their ability to develop into immunogenic effector T-cells, but stimulates development of regulatory T-cells that dampen the immune response^26^.

In agreement with the formation of steady-state DCs after phagocytosis of *A. terreus* conidia neither significant induction of the pro-inflammatory cytokine TNF-α, nor other cytokines was observed. This is in line with observations on *A. fumigatus*, in which β-glucan exposure from germinating conidia was required to trigger TNF-α production in bone marrow and alveolar macrophages as well as dendritic cells. In contrast, heat-killed *A. fumigatus* conidia were not immune stimulatory^27^ and in our experiments UV-inactivated *A. fumigatus* conidia behaved comparable in DC interactions as viable or UV-inactivated *A. terreus* conidia.

While persisting *A. terreus* conidia may cause a down-regulation of the immune response, they may also explain the difficulties to treat *A. terreus* infections with antifungals despite the fact that, except for AMB, antifungal susceptibility of *A. terreus* is largely comparable to that of *A. fumigatus* ^4^. It has been observed that significantly higher drug concentrations are required in therapy of *A. terreus* infections than assumed from *in vitro* susceptibility analyses and it virtually affects treatment with all common antimycotics^4^. This may, at least partially, be due to a fungistatic effect of drugs on *A. terreus*, which results in large differences between minimal inhibitory and minimum fungicidal drug concentration^28^. However, at 10 µg/ml of AMB or VOR both drugs showed high fungicidal activity on *A. terreus*, which was completely different in the presence of DCs. Within DCs between 40 and 60% of *A. terreus* conidia survived this drug treatment for the first 24 h and about 30% viable conidia were still re-isolated after three days. Therefore, persistence within DCs may not only tune down immune activation, but also supports evasion of *A. terreus* from antifungal treatment.

For using DCs as a vehicle for dissemination as frequently observed with *A. terreus* infections^6^, full maturation and expression of transmigration markers is required. As observed in moribund corticosteroid treated mice, *A. terreus* can indeed elicit a strong TNF-α response in infected lungs once hyphal elements are formed^13^. Alternatively, TNF-α production may be caused by bacterial (co-)infection as common in critically ill patients^29^. We have shown that external addition of TNF-α results in complete maturation and expression of CCR7 on DCs containing persisting *A. terreus* conidia, without resulting in increased conidia inactivation rates. TNF-α and IFN-γ activate production of reactive oxygen species in macrophages, which has been described to support clearance of intracellular fungal pathogens^8, 9^. However, in case of *A. terreus* infections, this appears of minor importance. A transient increase in IFN -γ in immunocompetent mice infected with *A. terreus* conidia has been observed^10^, which is in line with the Th1 immune response described for *A. terreus* infections^20^. However, as viable conidia remain persistent in infected lung tissues^10^, this clearance mechanism seems not effective for *A. terreus* conidia. Even more, the activation of DCs by these external cytokines may cause the initiation of transmigration to draining lymph nodes and spleen, transporting viable *A. terreus* conidia to organs outside the pulmonary tract. However, it needs to be mentioned that providing a definite role for the observed persistence of *A. terreus* conidia in dendritic cells during pathogenesis will require the *in vivo* detection of DCs carrying viable fungal elements.

In conclusion, MUTZ-3 cells are well suited for the investigation of *Aspergillus* conidia interactions with dendritic cells. Parallel studies on the survival of *A. fumigatus* and *A. terreus* conidia with MUTZ-3 DCs and moDCs showed identical results and our analyses on *A. fumigatus* conidia matched results previously obtained from primary or monocyte-derived DCs. As previously observed for macrophages, *A. terreus* follows a sit and wait strategy in DCs and may use these cells for dissemination once an external trigger stimulates DCs maturation. Most importantly, *A. terreus* was protected from antifungal treatment inside DCs, which could explain treatment failure despite *in vitro* susceptibility of *A. terreus* against antifungals^4^. Finally, we speculate that long-term persistence of *A. terreus* conidia within immune effector cells such as macrophages or dendritic cells may also occur in immunocompetent persons as previously observed in immunocompetent mice^10^. Thus, once an immunosuppressive regimen is applied these conidia may be released from immune cells and may cause invasive aspergillosis with high dissemination rates.

## Material and Methods

### Fungal strains, cultivation and preparation of conidia suspensions

*A. fumigatus* CBS 144.89 (CBS, Utrecht, Netherlands), *A. terreus* SBUG844 (HKI strain collection, Jena, Germany^30^) and *A. terreus* SBUG844 expressing the *A. nidulans w*A gene^10^ were used in this study. For conidia preparation, fungi were cultivated at 37 °C on solid 50 mM glucose containing *Aspergillus* minimal media with nitrate as nitrogen source and 2% agar^30^. *A. fumigatus* conidia were harvested after 3–4 and *A. terreus* conidia after 5–6 days of incubation. Conidia were harvested by overlaying plates with 10 ml sterile phosphate-buffered saline (PBS) and filtered through 40 μm cell-strainers. Conidia suspensions were washed twice in PBS and counted by using a haemocytometer.

### Labelling and UV-inactivation of conidia

For phagocytosis assays conidia were pre-labelled with fluoresceine isothiocyanate (FITC) as described previously^10^. In brief, 1–2 × 10^8^ conidia were suspended in 10 ml of 0.1 M sodium carbonate solution containing 0.1 mg/ml FITC and incubated at 37 °C for 1 hour. Conidia were washed twice in sterile PBS and diluted to selected concentrations for phagocytosis assay. For UV inactivation 1 ml of conidia suspension containing 2–3 × 10^8^ conidia were poured in small petri dish (5 cm in diameter). UV treatment was performed with the sterilisation programme of a GS Gene Linker UV chamber (BioRad Laboratories). Complete inactivation of conidia (>99.9%) was confirmed by spreading aliquots on malt extract agar plates.

### Differentiation of DCs from human monocytes

Human monocyte-derived DCs were generated as previously described^31^. In brief, human peripheral blood mononuclear cells (PBMCs) were harvested by density gradient centrifugation using Biocoll (Biochrom AG). CD14^+^ primary human monocytes were further purified from PBMCs by CD14^+^ microbeads (Miltenyi Biotech) using a magnetic cell sorting system. Primary monocytes were differentiated into monocyte-derived dendritic cells (moDCs) by treatment with 800 U/ml human recombinant GM-CSF and 1,000 U/ml human recombinant IL-4 (Miltenyi Biotech). At day six cell surface marker expression (Fig. S1A) and phenotype of immature moDCs was monitored prior to performing conidia interaction studies.

### Cultivation and differentiation of MUTZ-3 cells

The MUTZ-3 cell line derived from a human acute myelomonocytic leukaemia (DSMZ, Braunschweig, Germany) was kept in minimal essential alpha medium (MEM α, Gibco) supplemented with 20% of heat-inactivated fetal bovine serum (FBS) and 50 U/ml of recombinant human GM-CSF (Miltenyi Biotec). To differentiate MUTZ-3 cells into iDCs, 1–2 × 10^5^ cells/ml were incubated for 7 days in the presence of 300 U/ml human recombinant GM-CSF and IL-4 (Miltenyi Biotec)^22^. LSRII flow cytometry was applied to analyse expression of surface markers (Fig. S1B), using FITC-anti-human CD14, PE-anti-human CD1a, PE/Cy7-anti-human DC-SIGN and APC-anti-human CD83 (all BioLegend, London, UK). Differentiated iDCs were kept in MEM α medium supplemented with 20% of heat-inactivated FBS during *Aspergillus* conidia interaction studies.

### Phagocytosis assays

iDCs (3 × 10^5^ cells) were transferred to wells of a 24-well plate containing polylysine-coated coverslips (12 mm diameter) and FITC-labelled conidia of *A. fumigatus* or *A. terreus* were added at a multiplicity of infection (MOI) of 0.5. At selected time points plates were centrifuged at 200 × g to pellet cells and fixed over night at 4 °C in the presence of 4% of paraformaldehyde. After washing with PBS, cells were blocked for 15 min at room temperature (RT) with Fc receptor blocking solution (Human TruStain FcX^TM^, BioLegend). Plates were centrifuged again for 10 min at 200 × g before blocking solution was removed. To stain conidia not phagocytosed by DCs, a rabbit anti-conidia antiserum (kindly provided by Frank Ebel, Munich) diluted 1:100 in 1% bovine serum albumin (BSA, sigma) was added and incubated for 1 hour at RT. After washing with PBS, Cy3-labelled anti-rabbit IgG antibody diluted 1:300 in 1% BSA was added and incubated for 1 hour at RT in the dark. Coverslips were washed three times with PBS, mounted in ProLong Gold mounting solution (Invitrogen) and analysed by fluorescence microscopy. Conidia co-localising with DCs were counted. Phagocytosis rates were calculated from FITC^+^, Cy3^−^ conidia divided by the total number of conidia counted resulting in the percentage of phagocytosis. At least 900 conidia were analysed in each experimental group.

### Analysis of conidia germination

*A. terreus* and *A fumigatus* conidia (10^5^/well) were cultivated at 37 °C in a CO_2_ incubator in 96-well plates in the absence or presence of DCs (MOI = 1). Optical density of cultures (OD_600_) was analysed at 600 nm every hour. To investigate the effect of pH or nutrient limitation on germination of *Aspergillus* conidia, RPMI 1640 (Sigma) with 2% glucose was adjusted to pH 6–8 or MOPS-buffered medium (pH 6.5) was serially diluted with PBS. Cells were incubated at 37 °C in a Multiskan Go microplate spectrophotometer (Thermo) and OD_600_ was taken every 15 min. Similarly, *Aspergillus* minimal medium with ammonium chloride as nitrogen source was serially diluted and analysed for conidia germination. To analyse the effect of carbon or nitrogen starvation, either glucose or ammonium chloride were serially diluted, but all other components were kept constant.

### Killing and antifungal sensitivity of conidia in the presence of DCs

DCs (1 × 10^6^ cells/well) were co-incubated with *Aspergillus* conidia at an MOI of 0.1 in 6 well plates. DCs, including all extracellular fungal material, were scraped at 1 h, 3 h, 6 h and 9 h and lysed by the addition of sterile cold ddH_2_O with subsequent centrifugation at 2000 × g to release and collect intracellular conidia. Serial dilutions of cell lysates were plated on malt extra agar plates and incubated for 24–40 h at 37 °C for determination of CFUs. Survival rates were calculated with reference to CFUs from conidia not confronted with DCs. For antifungal sensitivity assays DCs were allowed to phagocytose *Aspergillus* conidia for 6 h prior to addition of 10 μg/ml AMB or VOR. DCs were collected and lysed as described after a total of either 24 h or 72 h. Serial dilutions of lysates were plated on malt extract agar plates and CFUs and survival rates were determined after 40 h of incubation at 37 °C as described above.

### Lysotracker staining

DCs and MH-S cells (3 × 10^5^ cells/well) were co-incubated with FITC-labelled *Aspergillus* conidia at an MOI of 0.5 on polylysine coated coverslips (12 mm) in 24 well plates. After 3 h plates were centrifuged at 200 × g for 10 min and medium was removed. Pre-warmed fresh medium containing 200 nM lysotracker (DND-99, Invitrogen) was added and incubation was continued for 1 h to allow for staining of phagolysosomes. After a brief centrifugation step, cells were fixed for 15 minutes with 4% paraformaldehyde. After washing with PBS, coverslips were mounted with ProLong Gold mounting solution and analysed by fluorescence microscopy.

### Analysis of DC activation markers and cytokine production

DCs (10^6^/well) were co-incubated with viable or UV-inactivated *Aspergillus* conidia (MOI = 2) in 6 well plates. For short-term activation studies cells were analysed after 6 h of co-incubation, whereas in long-term interaction studies amphotericin B (2 μg/ml) was added after 6 h to inhibit extracellular fungal growth. Cells were collected at 24 h and blocked for 10 min at RT with Fc receptor blocking solution (Human TruStain FcX^TM^). DCs (2–3 × 10^5^ cells) were treated with 1 μg/ml FITC-anti-human CCR7, APC-anti-human CD54, PE-anti-human CD80 or PerCP-anti-human HLA-DR (MHCII) monoclonal antibodies (BioLegend) diluted by FACS staining buffer (dPBS supplemented with 1% heat-inactivated FBS) and incubated for 30 min in the dark at 4 °C. After two washes in FACS staining buffer, cells were fixed in 1% paraformaldehyde and subjected to flow cytometry (LSRII). For preparation of intracellular cytokine staining, Brefeldin A (5 μg/ml, Fluka) was added 6 h before harvest to block intracellular protein transport. Cells were collected at 30 h and treated with Fc receptor blocking solution for 10 min at RT. Cells were fixed with 4% paraformaldehyde for 20 min at 4 °C. To make cell membranes permeable, fixed cells were washed with perm wash buffer (FACS staining buffer containing 0.1% saponin). The cells were stained at 4 °C for 30 min with 1 μg/ml of either PE-anti-human TNF-α, FITC-anti-human IL-1β, PE-anti-human IL-12/IL-23 p40, PE-anti-human IL-4, PE-anti-human TGF-β or APC-anti-human IL-10 monoclonal antibodies (BioLegend) prepared in perm wash buffer. After washing with perm wash buffer, the cells were left in 1% paraformaldehyde at 4 °C in the dark before analysed by flow cytometry. Mean fluorescence intensity (MFI) was analysed by FlowJo software and normalised to mock-infected DCs.

### Statistical analyses

Comparisons between multiple groups were analysed by one-way analysis of variance (ANOVA) followed by Tukey’s multiple comparison test using GraphPad Prism (GraphPad Software). Statistical differences between two groups were analysed by two-tailed Student’s t-test.

### Ethics statement

Human peripheral blood for generation of moDCs was collected from healthy volunteers after written informed consent. The study was conducted in accordance with the Declaration of Helsinki, all protocols were approved by the Ethics Committee of the University Hospital Jena (permit number: 273-12/09).

## Electronic supplementary material

Supplementary information
