# Supplementary material for: Persistence within dendritic cells marks an antifungal evasion and dissemination strategy of Aspergillus terreus
Source: Sci Rep. 2017 Sep 6;7:10590. doi: 10.1038/s41598-017-10914-w (PMC5587622; doi:10.1038/s41598-017-10914-w)
Supplement: Supplementary file 1 — Supplementary information [file 41598_2017_10914_MOESM1_ESM.pdf]

Supplementary information

Persistence within dendritic cells marks an antifungal evasion and dissemination strategy of *Aspergillus terreus*

Shih-Hung Hsieh, Oliver Kurzai, Matthias Brock

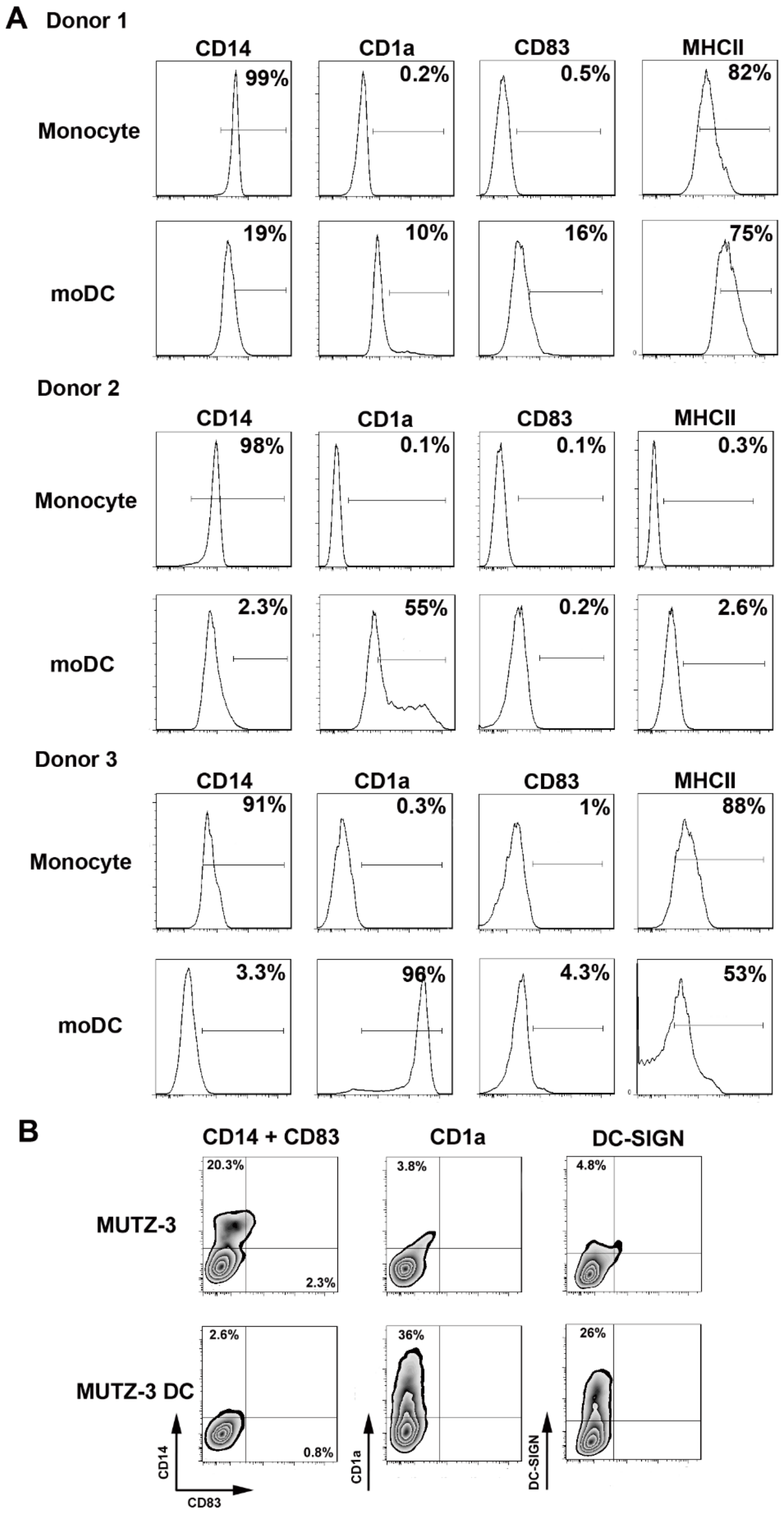

Figure S1: Analysis of DC differentiation from human peripheral blood monocytes and MUTZ-3 cells. (**A**) Flow cytometric analyses on the differentiation of DCs from human peripheral blood monocytes obtained from three different donors. All samples show a down-regulation of CD14 and up-regulation of CD1a with some variation among donor samples. (**B**) Representative flow cytometry analysis of the differentiation of immature DCs from MUTZ-3 cells. CD14 is significantly down-regulated, whereas expression of CD1a and DC-SIGN increases during differentiation.

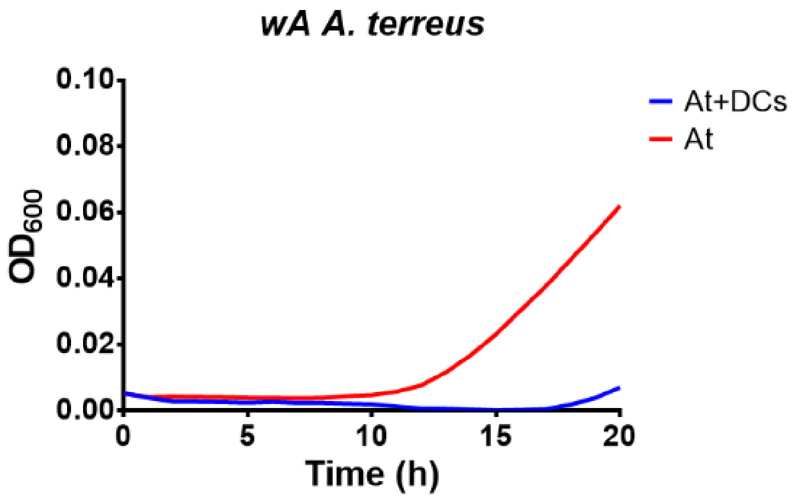

Figure S2: Escape of *A. terreus* conidia expressing the naphtopyrone synthase *wA* from *A. nidulans* from MUTZ-3-derived iDCs. *wA* expression does not increase escape of *A. terreus* conidia from iDCs. Growth curves represent mean values from three independent experiments. For comparison with wild-type *A. terreus* conidia refer to Fig. 1D in the main manuscript.

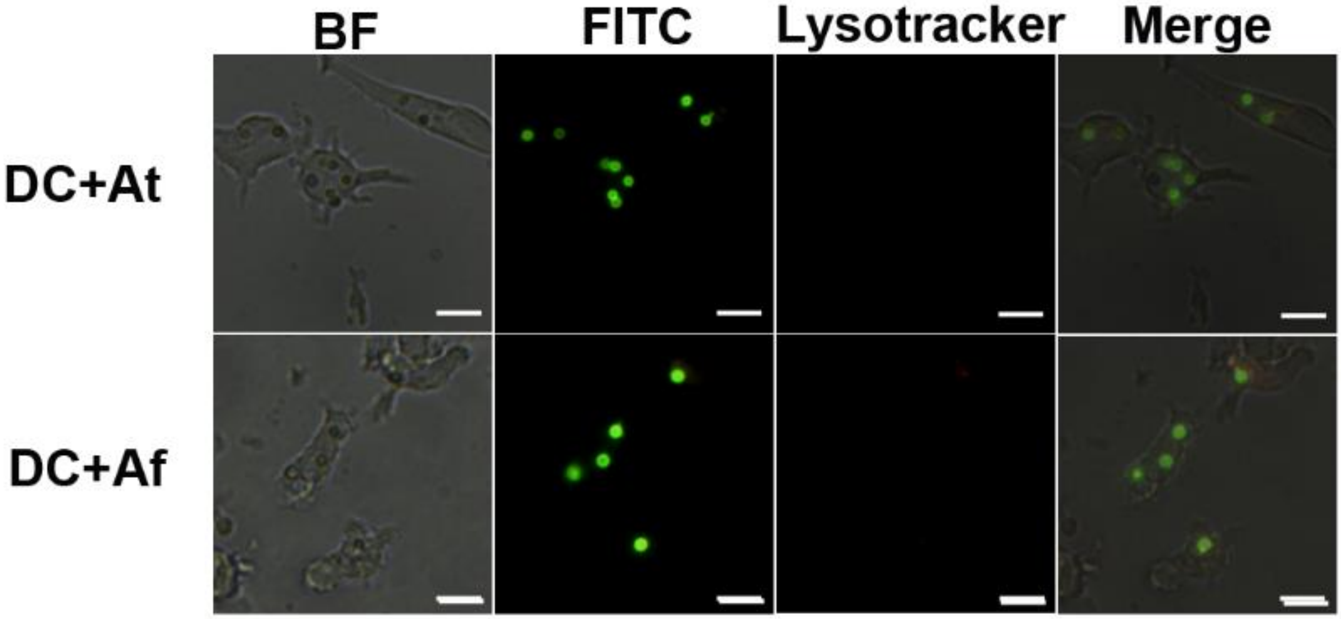

Figure S3: Analysis of phagolysosome acidification of DCs after phagocytosis of *A. fumigatus* and *A. terreus* conidia. MUTZ-3-derived iDCs were incubated with FITC-labelled *A. terreus* (At) or *A. fumigatus* (Af) conidia at an MOI of 0.5. After 3 h of co-incubation lysotracker DND-99 staining was performed and cells were analysed by fluorescence microscopy. Scale bar = 10 µm; BF = bright field. No lysotracker staining is observed that co-localises with intracellular conidia.

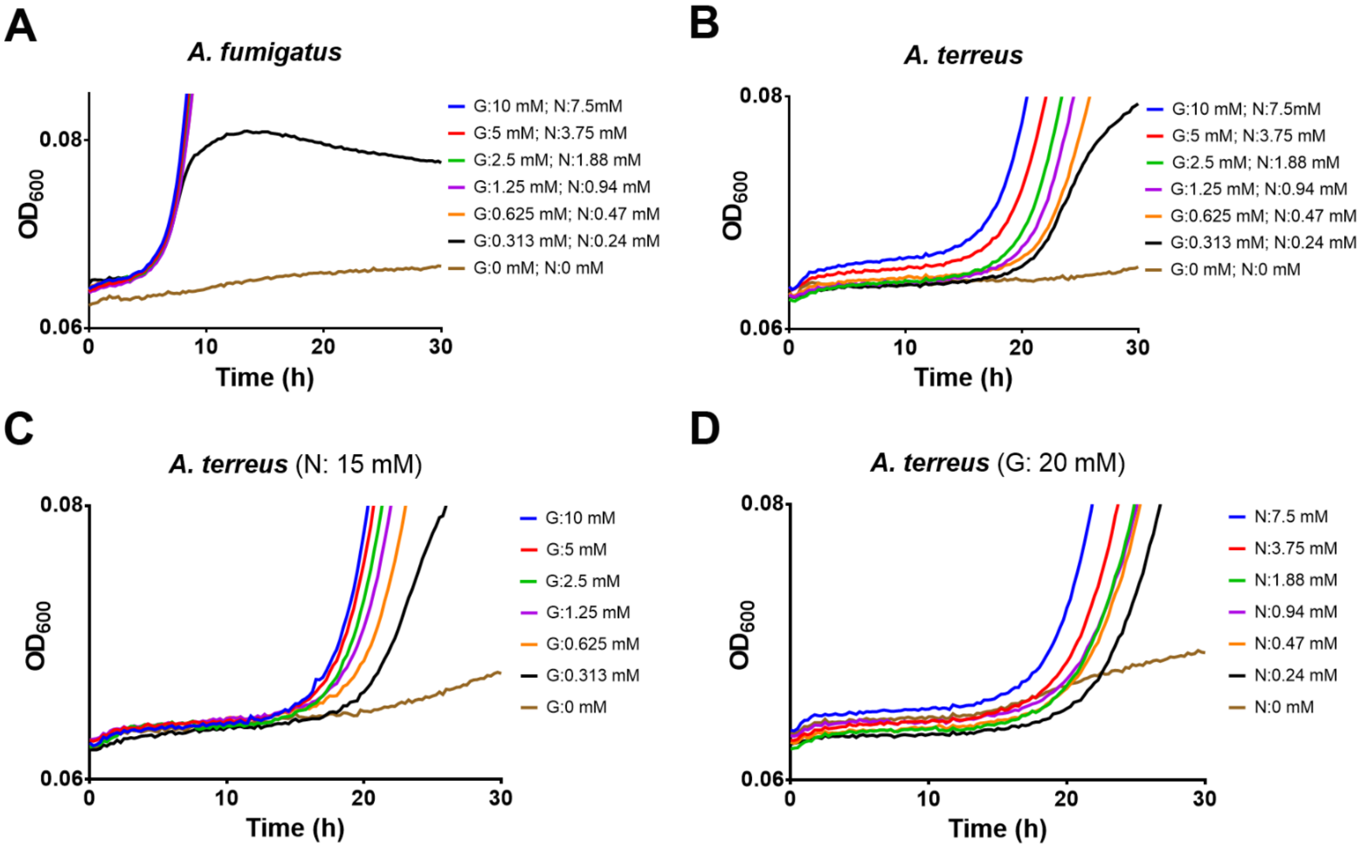

Figure S4: Impact of nutrient limitation on germination of *A. fumigatus* and *A. terreus* conidia. (**A**, **B**) *Aspergillus* minimal medium containing 10 mM glucose as carbon and 7.5 mM ammonium chloride as nitrogen source were serially diluted and inoculated with (A) *A. fumigatus* or (B) *A. terreus* conidia. Initial germination was analysed by monitoring the increase in optical density at 600 nm (OD_{600}). Even at low carbon and nitrogen concentrations *A. fumigatus* shows no delayed germination, whereas *A. terreus* germination speed depends on nutrient availability. (**C**) Effect of nitrogen and (**D**) glucose limitation on germination speed of *A. terreus* conidia. Both limitations provoke a germination delay. Growth curves show mean values from two independent experiments, each performed in technical triplicates.

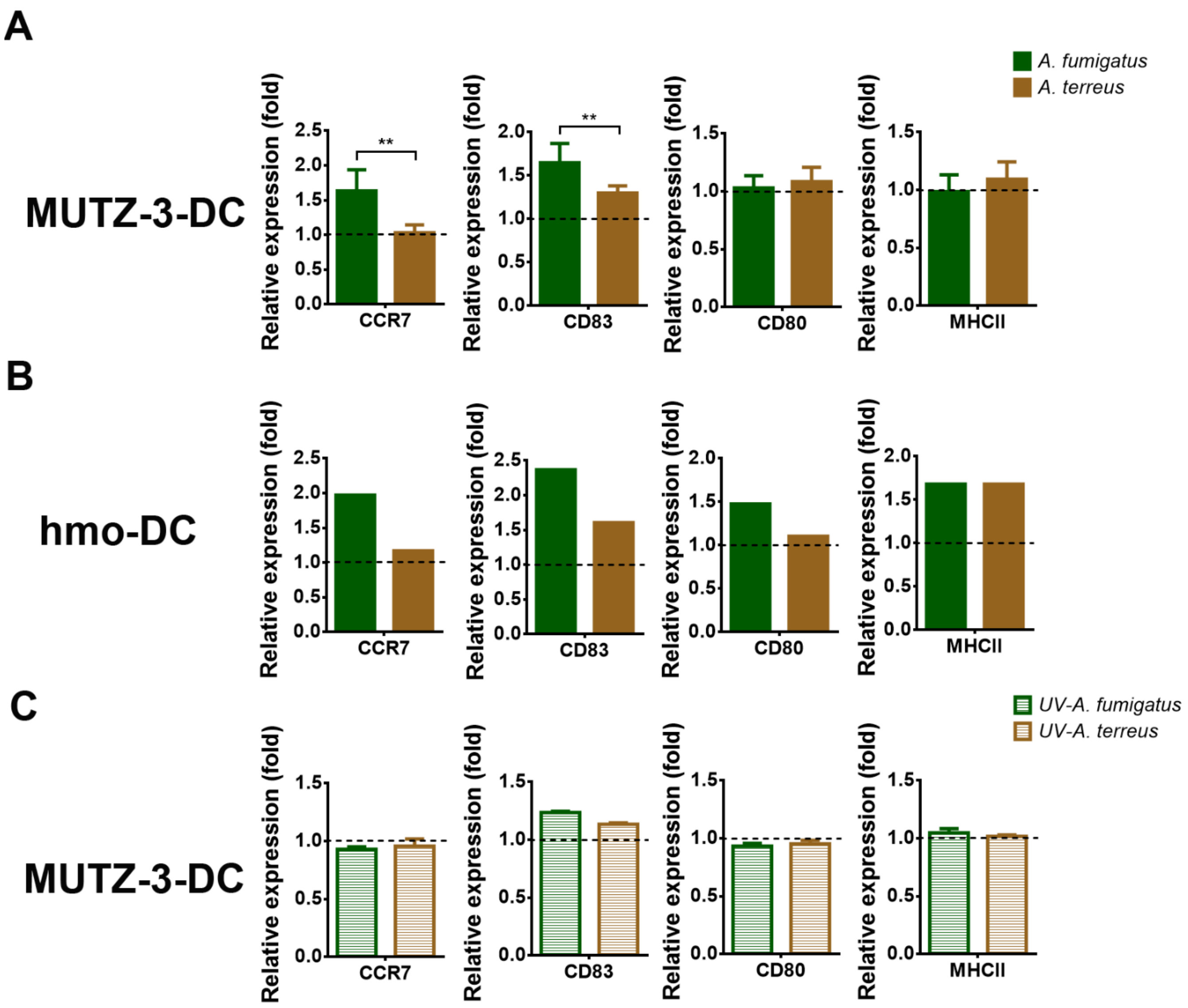

Figure S5: Expression of DC activation markers after short-term interaction with *A. fumigatus* and *A. terreus* conidia. For analysis of activation and maturation markers (CCR7, CD83, CD80 and MHC II), MUTZ-3-derived iDCs or human monocyte-derived DCs (hmo-DC) were incubated for 6 h with *A. fumigatus* or *A. terreus* conidia. Expression levels were normalized to uninfected DCs (dashed line). (**A**) Marker expression of MUTZ-3-DCs confronted with viable *A. fumigatus* and *A. terreus* conidia. (**B**) Marker expression of hmo-DCs confronted with viable *A. fumigatus* and *A. terreus* conidia. (**C**) Marker expression of MUTZ-3-DCs confronted with UV-inactivated *A. fumigatus* and *A. terreus* conidia. Data in (A) and (C) represent mean values + SD from three independent experiments and statistics were performed by one-way ANOVA (** $p < 0.01$). Data in (B) represent mean values from two independent experiments and indicate the same trend as observed for MUTZ-3 cells.
